# Veterinary Medicine Needs New Green Antimicrobial Drugs

**DOI:** 10.3389/fmicb.2016.01196

**Published:** 2016-08-03

**Authors:** Pierre-Louis Toutain, Aude A. Ferran, Alain Bousquet-Melou, Ludovic Pelligand, Peter Lees

**Affiliations:** ^1^Ecole Nationale Vétérinaire de Toulouse, Institut National de la Recherche Agronomique, TOXALIM, Université de ToulouseToulouse, France; ^2^Comparative Biomedical Sciences, The Royal Veterinary CollegeHatfield, UK

**Keywords:** innovative eco-friendly antimicrobials, veterinary medicine, public health, commensal microbiota, environmental hazard

## Abstract

Given that: (1) the worldwide consumption of antimicrobial drugs (AMDs) used in food-producing animals will increase over the coming decades; (2) the prudent use of AMDs will not suffice to stem the rise in human antimicrobial resistance (AMR) of animal origin; (3) alternatives to AMD use are not available or not implementable, there is an urgent need to develop novel AMDs for food-producing animals. This is not for animal health reasons, but to break the link between human and animal resistomes. In this review we establish the feasibility of developing for veterinary medicine new AMDs, termed “green antibiotics,” having minimal ecological impact on the animal commensal and environmental microbiomes. We first explain why animal and human commensal microbiota comprise a “turnstile” exchange, between the human and animal resistomes. We then outline the ideal physico-chemical, pharmacokinetic, and pharmacodynamic properties of a veterinary green antibiotic and conclude that they can be developed through a rational screening of currently used AMD classes. The ideal drug will be hydrophilic, of relatively low potency, slow clearance and small volume of distribution. It should be eliminated principally by the kidney as inactive metabolite(s). For oral administration, bioavailability can be enhanced by developing lipophilic pro-drugs. For parenteral administration, slow-release formulations of existing eco-friendly AMDs with a short elimination half-life can be developed. These new eco-friendly veterinary AMDs can be developed from currently used drug classes to provide alternative agents to those currently used in veterinary medicine and mitigate animal contributions to the human AMR problem.

## Introduction

The links between animals and humans, in respect of the emergence and spread of resistance, is a major global issue. This review proposes the development for veterinary medicine of new and innovative drugs for food-producing animals; this is not for animal health reasons but rather to mitigate the veterinary contribution to the human resistome.

Currently, food-producing animal medicine does not face the same critical situation as human medicine, because there are neither life-threatening infections of multiply drug resistant microorganisms causing sepsis nor chronic conditions in poultry, pigs, or cattle for which AMD therapy is mandatory. In addition, the prevalence of resistance for major veterinary pathogens that cannot be treated by any AMD is very limited. However, this review proposes a renewal of the veterinary armamentarium with drugs designed to break, for public health reasons, the link between human and veterinary medicine. We have termed, these innovative compounds “green antibiotics,” as they will have minimal (ideally no) ecological impact on the animal commensal microbiome and, more broadly, on the environmental resistome (Toutain and Bousquet-Melou, 2013; Toutain et al., 2015).

The rationale for and urgency of this proposal is that (i) the worldwide consumption of AMDs to treat or prevent health conditions in food-producing animals will ineluctably increase over the coming decades (Van Boeckel et al., 2015) (ii) the so-called prudent use of AMDs will not stem the rise in AMR of animal pathogens and commensals and its subsequent impact on the human resistome.

New, eco-friendly, veterinary AMDs can readily be developed from currently used drug classes to provide credible alternative agents. The viability of this approach is enhanced by the fact that alternatives to AMD use are either not available or not implementable as reviewed by Cheng et al. (2014). The green antibiotic principle is in line with the so-called eco-evo concepts that consider AMR in the broad light of evolution and ecology, rather than that of narrow clinical practices relating to infections (Baquero et al., 2011). In this review, we first consider the animal and human commensal microbiota as comprising a “turnstile” for exchanges between the two resistomes, and then outline the ideal properties of a green antibiotic. Finally, the regulatory aspects that should be addressed to facilitate the promotion of green antibiotics will be discussed.

## One World, One Health and “One Resistome”

In adhering to the principle of *One world, One health* (i.e., acknowledging the interconnections between animal and human health and the environment) the priorities for veterinary AMD therapy are dictated by public health rather than animal health issues. The tonnage consumption of AMDs in veterinary medicine exceeds that of human medicine (World Health Organisation, 2012), and it is recognized that veterinary medicine contributes to the emergence and spread of AMR in humans. Available epidemiological methods alone are often insufficient to accurately describe the relationships between agricultural AMD use and resistance (Singer and Williams-Nguyen, 2014). Therefore, the veterinary contribution to human AMR remains uncertain, with opinion ranging from globally negligible (Phillips et al., 2004) or irrelevant [an example is resistance to fluoroquinolones in *Escherichia coli* and non-typhoidal *Salmonella* zoonosis (de Jong et al., 2012)], to one of a major concern (Collignon et al., 2013). Despite this division of opinion, it is clear that AMD uses in livestock play some role in the emergence, amplification, persistence and transfer of resistance determinants to all ecosystems (Marshall and Levy, 2011) and the main justification in promoting the concept of green antibiotics is to minimize the veterinary contribution to the enrichment of human and environmental resistomes.

## What Types of Antimicrobial Drug Resistance Does Veterinary Medicine Face and Which Raise Public Health Issues?

**FIGURE 1:**
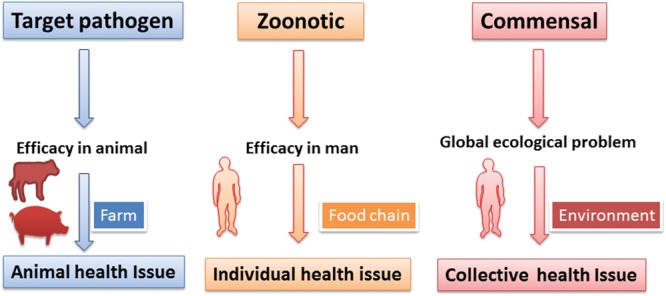
**Classes of bacteria developing resistance under the influence of veterinary AMD usage and consequences for animal, human and public health.** The veterinary use of AMDs is inescapably linked to the risk of emergence of AMR in veterinary targeted pathogens, zoonotic food-borne pathogens and the animal commensal microbiota. AMR for the targeted pathogen raises animal health issues only, whereas AMR for zoonotic pathogens and bacteria in the commensal microbiota (principally in the digestive tract) raises public health issues. However, for zoonotic pathogens, the problem, although important, is solely an individual medical problem. For the animal commensal microbiota, on the other hand, the concern is ecological, collective and worldwide. It is this latter issue that drives the requirement for new, green AMDs in veterinary medicine.

Veterinary medicine faces AMR of three types: AMR for specific animal pathogens; AMR for zoonotic pathogens; and AMR of the commensal bacteria harbored by animals (**Figure 1**).

Antimicrobial resistance of specific animal pathogens raises veterinary problems in terms of efficacy but, for two reasons, has no direct impact on human medicine: (i) these pathogens (resistant or not) are not in most instances zoonotic and (ii) more importantly, the size of these pathogenic microbiota is negligible, when compared to the size of the commensal microbiota that are collaterally exposed during AMD treatment (see later).

Antimicrobial resistance of foodborne zoonotic pathogens, causing enteritis and diarrhea, such as *Salmonella (Salmonella typhimurium and S. enteritidis), Campylobacter jejuni (in poultry), C. coli (in pigs)* and some strains of *E. coli such* as *E. coli O157:H7* are of greater concern. This was the case for the in ovo administration of ceftiofur, an emblematic example of AMD misuse in hatcheries, with the aim of improving the sanitary status of chickens (Dutil et al., 2010). However, the emergence of AMR in foodborne pathogens is not the most relevant hazard of veterinary origin for human medicine. According to a recent EFSA report on human cases of salmonellosis and campylobacteriosis in the EU (European Food Safety Authority, 2014b), approximately 200 deaths were attributable annually to these zoonoses. This number is placed in perspective by the claimed 25,000 deaths attributed to AMR annually in the EU (World Health Organisation, 2015). Similar figures were reported in the USA (Centers for Disease Control and Prevention, 2013). Moreover, AMR *per se* is not responsible for these fatalities, as most zoonotic *Salmonella* and *Campylobacter* of EU foodborne origin are susceptible to the drugs available to treat these infections (European Food Safety Authority, 2014a). Furthermore, most cases of salmonellosis and campylobacteriosis in humans are self-limiting, not requiring antimicrobial treatment. Moreover, outbreaks of salmonellosis, at least in the EU, are decreasing; a 30% decline has been reported over the past 5 years (European Food Safety Authority, 2014b). Therefore, it can be concluded that resistance to zoonotic pathogens is an individual person medical issue and not a global ecological and economic hazard for the future.

**FIGURE 2:**
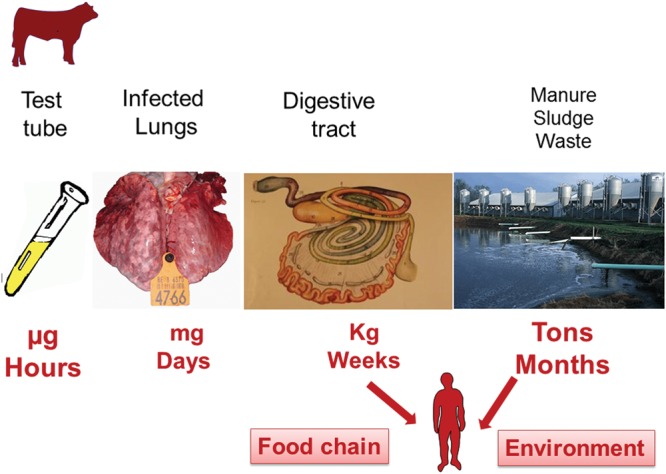
**Bacterial load exposed to AMDs during and after treatment, and the duration of exposure.** One important category of bovine respiratory disease is pasteurellosis. Roof (2011) estimated the pathogen load for the entire lung to be 2 × 10^7^–2 × 10^8^ colony forming units (CFU) for *Pasteurella multocida* and 9 × 10^6^–9 × 10^8^ CFU for *Mannheimia haemolytica*. Therefore, the estimated pathogen biomass in the lung of an infected cow does not exceed a few mg in toto, whereas the bacterial mass in the animal’s commensal GIT microbiota amounts to several kg. The duration of exposure of the target pathogen can be nil (prophylactic use) or very short (with rapid pathogen eradication during metaphylaxis). In contrast, the duration of intestinal microbiota exposure will never be less than the duration of treatment plus the delay in fully clearing the AMD, together with any newly formed resistant bacteria from the intestinal microbiota, i.e., several weeks (Hansen et al., 2002). The GIT microbiota are continually eliminated at a high rate into the environment, often into an aqueous matrix, thereby allowing further dissemination of the excreted and potentially resistant bacteria. Furthermore, this process will favor horizontal exchanges of resistance factors between organisms within this vast ecosystem. In consequence, this pathway of bacterial elimination (together with their genes) via the excreta is by far the largest connection between animal and human resistomes.

The hazards associated with AMR at the level of the animal’s commensal microbiota, i.e., organisms of the GIT and possibly of the skin are potentially much more serious ecologically. This is because their biomasses greatly exceed those of the specific or zoonotic pathogens harbored by the same treated animals (**Figure 2**). It is likely that the amplification of pre-existing or emerging genes of resistance displays some proportionality with both the size and genetic richness of each category of microbiota, whether pathogenic or not, harbored by treated animals. The commensal microbiota bacteria are not pathogenic but they ineluctably harbor, even before any antimicrobial treatment, a range of genes of resistance (the so-called resistome). The use of veterinary AMDs can promote the selection and amplification of the GIT resistant genes, which may then be transmitted directly (principally by the food chain) or indirectly (via the environment) to man. After gaining access to the human GIT microbiota, these “Trojan horse” bacteria may transmit their resistance genes to the human commensal bacteria by horizontal transfer; these genes of resistance may then be acquired by human-specific pathogens (Angulo et al., 2004) or by opportunistic bacteria such as *Enterococcus* spp. responsible for nosocomial infections.

To assess this collateral risk of AMD use in veterinary medicine, AMR in commensal bacteria is being monitored in indicator organisms of food-producing animals, *E. coli* for the Gram negative microbiota and *Enterococcus (Enterococcus faecium* and *E. faecalis*) for the Gram-positive commensal intestinal microbiota (European Food Safety Authority, 2013). For commensal *E.coli*, the link between the quantities of the different classes of AMDs administered to food-producing animals in EU and the prevalence of resistance in isolates from cattle, pigs, and poultry was demonstrated (Chantziaras et al., 2014). These epidemiological data drive the proposal that both human and veterinary medicine will benefit from the development of green AMDs by limiting the impact of AMD treatment on the animal commensal resistome and thence on that of humans.

## Shortcomings of the Paradigm of Prudent Use of AMDs in Veterinary Medicine

Many recommendations have been made on the prudent use of AMDs in livestock, to mitigate the emergence of AMR by promoting their sustainable use in animals. The most effective decisions, *a priori*, are those that enforce drastic reductions in the overall consumption of AMDs. However, the reduction of such use has, in some cases, produced unexpected results. For tetracycline resistance in fecal coliforms isolated from swine, the decrease was less than 50%, after the use of all classes of AMD had been discontinued for 126 months (Langlois et al., 1983). In the USA, the decision in 2005 to ban enrofloxacin for metaphylactic use in poultry was not followed by the expected decrease in AMR for *Campylobacter*. Indeed, by 2010 the prevalence of ciprofloxacin-resistant *C. jejuni* remained >20% for poultry and human clinical isolates (Food and Drug Administration, 2012).

Among the factors explaining the limited efficiency of a ban or of voluntary restriction of AMD use, the most challenging, is of ecological origin. When a wild-susceptible bacterial population has been replaced in the environment by an antimicrobial-resistant population, having no or low fitness costs associated with the mechanism of resistance, the emergent resistant population can become very stable in its ecosystem (Andersson and Hughes, 2010). Indeed, it was shown for *Campylobacter* in the USA that some mutations conveying resistance to ciprofloxacin might even provide a fitness advantage (Luo et al., 2005; Zhang et al., 2006). The general conclusion from bans and moratoriums is that retrospective measures will be less effective than tackling the factors leading to AMR emergence and dissemination in the first instance.

**FIGURE 3:**
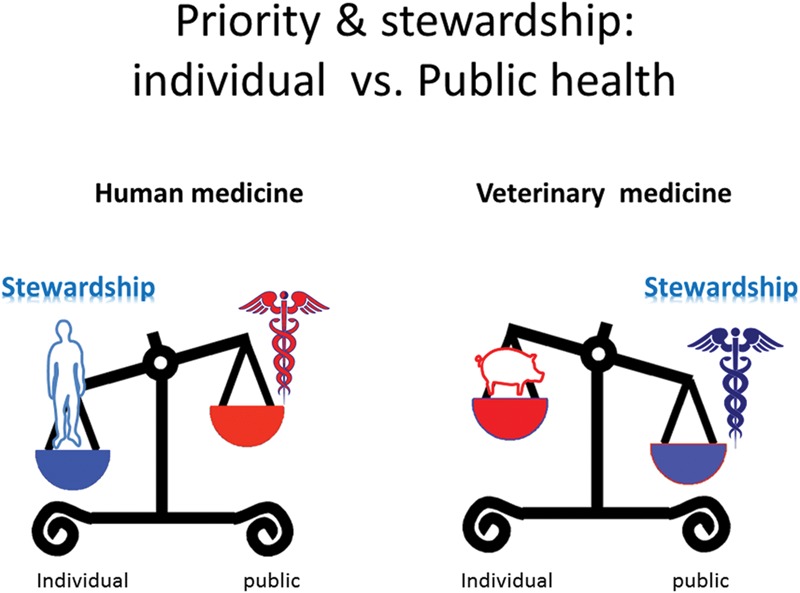
**Priority and stewardship for human and veterinary medicine and the paradigm of prudent use of AMDs.** The paradigm of prudent use of AMDs in animals can be insufficient and even counter-productive. This is because such recommendations fail to recognize that the main sources of the resistance determinants, which are amplified by veterinary AMD usage, are derived not from the pathogenic microbiota but from the commensal GIT microbiota. An appropriate stewardship regarding the target pathogen (the priority for human medicine) can actually increase the public health issues when directly extrapolated to veterinary medicine. For example, one recommendation, for the prudent use of AMDs in human and animal medicine, fully justified from both animal and human health perspectives, is the possible increase of dosage regimens for older drugs to comply with current PK/PD concepts. However, this may be detrimental from the perspective public health. A second example of a questionable recommendation is the compulsory recourse to Antimicrobial Susceptibility Testing for AMDs classified as critical for human use, when, in most instances, there are no specific corresponding veterinary breakpoints. Another issue is the marketing of inexpensive generic products in veterinary medicine. Although these have important uses in disease control, there is the possibility that they might be used clinically when (more costly) hygienic, husbandry and disease containment options would be more appropriate [for details see (Toutain and Bousquet-Melou, 2013)]. An *a priori* sound recommendation is to give preference to local rather than systemic AMD administration, as in the treatment of clinical mastitis or at drying off in dairy cattle. In fact this may be counterproductive as it does not allow for the fact that the waste and unsaleable milk (containing a higher residual amount of AMD than that associated with systemic treatment), is commonly used to feed calves and may be responsible for the emergence of resistance in their GIT microbiota (Brunton et al., 2012; Duse et al., 2013).

When the use of AMDs in animals is justified by welfare and economic considerations, veterinary prescribers have been encouraged to follow guidelines to ensure their so-called “prudent” use. Unfortunately, many recommendations have simply been transposed from human to veterinary medicine, without recognition that they may be inefficient and even counterproductive in respect of public health (**Figure 3**). As indicated above, the microbiota of public health interest are the animal GIT microbiota and ultimately the microbiota in the environment, rather than the target pathogen. For example, there is no certainty that the priority given to using older AMD classes, such as tetracyclines, qualified by EMA as category 1 (lower risk) drugs (European Medicine Agency, 2014) will be less detrimental to the human and environmental resistomes than, for example, a third generation cephalosporin, specifically developed to have minimal impact on the GIT microbiota and which is rapidly degraded in the environment. Indeed, the poor oral bioavailability of tetracyclines in food-producing animals is a factor leading to extensive animal GIT and environmental exposures. Resistance to tetracyclines is commonly associated with multi-drug resistant bacteria, able to co-select genes conferring resistance to highly critical AMDs for man (Herrick et al., 2014), despite the fact that these AMDs are not marketed or legally restricted in use for food-producing animals.

## The Discovery and Implementation of Alternatives to AMDs

Superficially attractive alternatives to AMDs include vaccines, antibodies to specific pathogens, immunomodulatory agents, bacteriophages, antimicrobial peptides and pro-, pre-, or symbiotic products. An example is the marked reduction of AMD consumption in Norway, following the marketing of an efficacious vaccine for the prevention of furunculosis (Midtlyng et al., 2011). Similarly, the use of a vaccine to prevent diarrhea due to *Lawsonia intracellularis* in pigs led to the reduction of AMD consumption in Danish pigs (DANMAP, 2014). However, for biological, technical, economic, medical and regulatory reasons, vaccines (like many putative alternatives to antibiotics) may be difficult to develop in veterinary medicine [reviewed by (Cheng et al., 2014)]. Moreover, some AMD alternatives can have negative consequences for public health, including the unexpected promotion of AMR. For example, food supplementation with trace elements, such as Zn and Cu, proposed as alternatives to AMDs to control colibacillosis in pigs (Fairbrother et al., 2005; Hojberg et al., 2005) increased the proportion of multi-resistant *E. coli in vivo* in the enteral microbiome of pigs (Bednorz et al., 2013) and also increased resistance to methicillin in staphylococci (reviewed by Yazdankhah et al., 2014).

## Which Animal Bacterial Ecosystems Promote AMR of Human Health Relevance?

The animal bacterial ecosystems possibly exposed to AMDs during treatment, and able to promote AMR of human health relevance, must be identified. In this review, three types of microbial ecosystems are considered, based on the two main hazard factors for the emergence and spread of resistance. The factors are their biomass and the link of each system with the environment: (i) large, open bacterial ecosystems, such as the GIT and skin microbiota; (ii) small, open ecosystems, such as the respiratory tract; and (iii) small, closed ecosystems, such as the udder in cattle. The healthy udder is a closed system with no resident flora and is unable to foster a significant source of AMR during systemic AMD administration. The lung is an open system with no relevant resident microbiota and the bacterial biomass in the lung exposed to AMD during a lung infection is very small, not exceeding a few mg (**Figure 2**). In contrast, considering the estimated total numbers of prokaryotes in the GIT of some domestic species (Whitman et al., 1998) and assuming an average weight of 1 pg per prokaryote cell, the bacterial biomasses in the digestive tract of a typical pig, cow, chicken, and man are approximately 500, 3,000, 0.2, and 70 g, respectively. Thus GIT biomass is several orders of magnitude greater than that of the target lung pathogens. In addition, the GIT microbiota contain a large genetic diversity, including many resistance genes that can be amplified, and they have a long residence time in the GIT, favoring exchanges of resistance genes. There is, moreover, a regular large scale release of this bacterial population into the environment, thereby potentially impacting on the emergence and/or selection of AMR.

Sludge and manure are waste products exposed to AMDs or their active metabolites, not only during antimicrobial treatments but also long after its completion; the bacterial biomass exposed to AMDs is expressed in tons, not mg as for the target pathogen or kg for the commensal microbiota (**Figure 3**). Indeed, in cattle feces production rates are 12 kg/day for calves, 26 kg/day for beef and 62 kg/day for milk cows. In pigs, the daily production of manure is 1 to 4 kg and for egg-laying poultry it is approximately 100 g (Hofmann, 2008). In consequence, the risk, when treating a pulmonary infection in domestic species, is not due to AMD exposure of the targeted pathogens but to the unwanted exposure of the intestinal microbiota to the administered drug and beyond, to the persistency of its biological effects in soil and water bacterial populations (van den Bogaard and Stobberingh, 2000; O’Brien, 2002).

## Why Veterinary Antimicrobial Treatments are Able to Alter the Resistome of the Animal Git Microbiota

**FIGURE 4:**
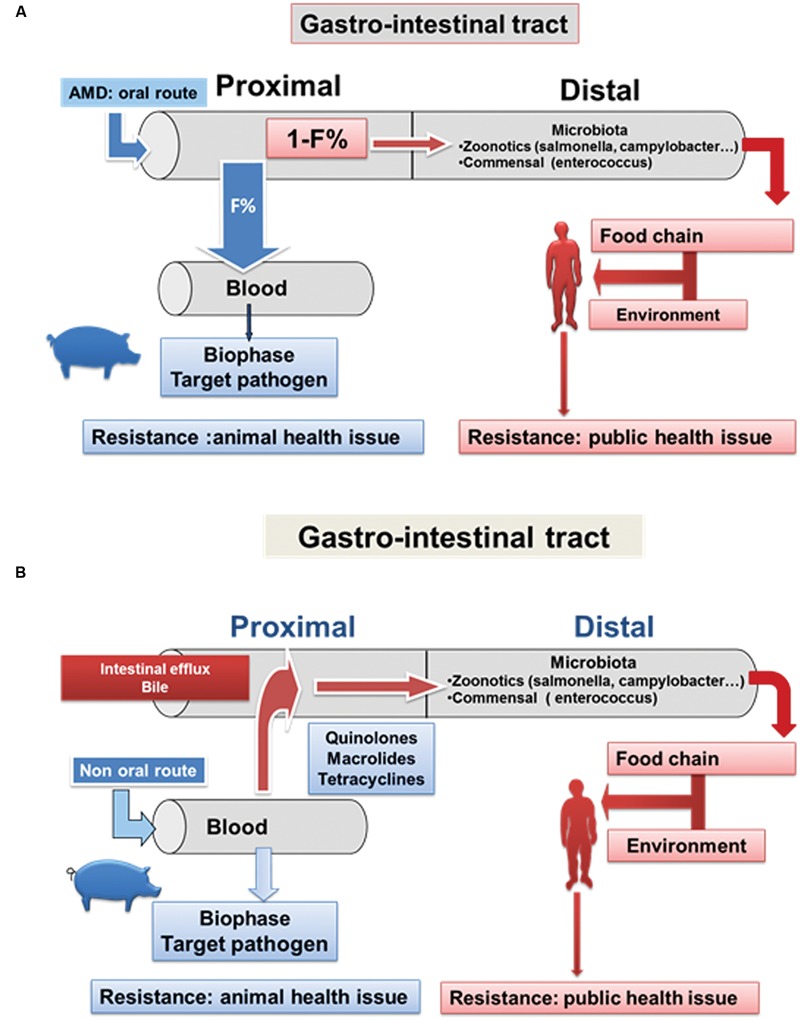
**Impact of AMD administration on the Gastrointestinal (GIT) microbiota after oral and parenteral administration. (A)** After oral AMD administration, the distal intestinal microbiota (comprising zoonotic pathogens and the commensal flora) is exposed to the unabsorbed drug fraction in the proximal segment of the digestive tract (1-F%, where F = bioavailability). Potentially, this can lead to local selective pressure, thereby increasing the density of the resistant bacteria and resistance genes. This load of enriched resistant bacteria and resistance genes is then released into the environment via fecal excretion. These organisms and genes can reach humans by several pathways and thereby ultimately gain access to the human GIT microbiota. The principal pathway is via the food chain. **(B)** After systemic AMD administration most drugs used in veterinary medicine are eliminated, to varying degrees but often extensively, in the digestive tract, either by biliary secretion or by intestinal clearance as a consequence of eﬄux pumps in the GIT wall. Intestinal drug concentrations capable of selecting for resistant organisms, with consequential detrimental effects, may occur, as for the oral administration route. In addition, the impact of AMDs on the animal GIT microbiota is not limited to the easily cultivable aerobic fraction, which accounts for only 1–2% of the total microbiota (typically *Escherichia coli* and *E. faeciens* which are considered as sentinel bacteria). AMDs can also negatively impact on the much larger anaerobic population [reviewed by (Holman and Chenier, 2015)].

In food-producing animals, the most convenient route of AMD administration is orally (**Figure 4**); this allows collective treatment at the herd or flock level for prophylaxis or metaphylaxis. Metaphylaxis also termed control in US correspond to the collective treatment of all animal of a group when only a given percentage of subjects of this group display the first signs of infection while prophylaxis is the administration of antibiotic when only a risk factor is present (weaning in piglet, transportation in calves, drying off in dairy cattle…).

The classes of oral AMDS most commonly used in food-producing animals are tetracyclines, β-lactams and sulphonamides. Tetracyclines (tetracycline, chlortetracycline, oxytetracycline and doxycycline) have very low oral bio-availability in pigs, with values typically between 5 and 15% (Pijpers et al., 1991; Nielsen and Gyrd-Hansen, 1996). In poultry, bioavailability is even lower, generally not exceeding 5% (Pollet et al., 1983). The unabsorbed drug fraction (85–95%) transits into the distal sections of the GIT (caecum, colon) exposing the body’s densest bacterial population for a duration that commonly exceeds treatment duration (Hansen et al., 2002). Thereafter, the unabsorbed fraction is excreted in feces into the environment as microbiologically active compounds. The oral bioavailability for ampicillin in pigs is also very low (10%), accounting for an altered microflora within 24 h after a single dose, and requiring several weeks before return to the control microbiota organisms (Bibbal et al., 2007).

Exposure of GIT microorganisms may also be high after systemic administration (intramuscular, subcutaneous injection) as most drugs used in veterinary medicine are to varying degrees eliminated by the digestive tract via intestinal eﬄux pumps as *P*-glycoprotein for quinolones (Alvarez et al., 2008). It may be noted that large fractions of the *Campylobacter C. jejuni* and *C. coli* populations adhere directly to the intestinal brush border (Naess et al., 1988) precisely where these eﬄux pumps are located. The consequence is a local high concentration in the mucosa-associated mucus layer, reported to be a site for the development of resistance to fluoroquinolones (Stamey and Bragonje, 1976).

After systemic administration most veterinary AMDs can attain intestinal concentrations capable of inhibiting intestinal bacteria and selecting resistant organisms. For example, it was shown that after intramuscular administration of ampicillin in pigs (20 mg/kg for 7 days), excretion of the Bla-TEM gene was immediately increased after the first dose (Bibbal et al., 2007). Quinolones are also extensively excreted into the GIT. After intravenous administration of danofloxacin in healthy pigs, high concentrations were present in all parts of the intestinal tract, resulting in AUC content-to-plasma ratios between 52⋅4:1 and 99⋅4:1 (Lindecrona et al., 2000). Foster et al. (2016), using ultrafiltration devices implanted in the GIT lumen, measured free enrofloxacin (active fraction) concentrations. The AUC intestinal fluid-to-plasma ratios were 1.6:1 and 2.5:1 in the ileum and colon, respectively. The intestinal concentrations were shown to be bactericidal for *S. enterica* and able to significantly inhibit *E. faecalis*.

Cephalosporins are more hydrophilic than fluoroquinolones and the lower intestinal exposure expected after parenteral administration has been verified after a subcutaneous ceftiofur administration. The penetration ratios were 0.39:1 and 0.25:1 for the ileum and colon, respectively, suggesting that ceftiofur is less extensively intestinally excreted than fluoroquinolones(Foster et al., 2016).

Macrolides are also excreted into the GIT but their local action should take into account the pH dependency of their antimicrobial activity. The pH in the colon contents of growing beef cattle and pigs is usually less than 7.0, which is likely to decrease macrolide potency. This might explain the lack of antimicrobial activity of tildipirosin in the GIT, after administration to beef cattle and pigs, for against foodborne pathogens and commensal bacteria (Rose et al., 2016).

## Elimination of AMDs in the Environment and Impact on Environmental Microbiota

It is acknowledged that the selection of AMD-resistant bacteria in the environment could jeopardize human health (Ashbolt et al., 2013; Wellington et al., 2013). Many of the known resistance factors of clinical concern have been recruited from non-pathogenic environmental bacteria (Bonomo and Szabo, 2006).

Sources of the AMDs contaminating the environment include food-producing animals excreting active compounds in feces and/or urine with the potential for exerting selective pressure on the microbiota in waste, sludge and manure (Heuer et al., 2011) and thereafter in the matrices of the environment (water, soil.) [reviewed by (Sarmah et al., 2006)]. In addition, several AMDs remain stable in the environment for several weeks or even months (Thiele-Bruhn, 2003).

The environmental pathways of AMR exchanges between animals and humans reveal new opportunities to mitigate the proliferation of resistant bacteria by appropriate management of human and agricultural waste for the currently used AMDs and highlight advantages of green AMDs which are either excreted as inactive metabolites or rapidly inactivated in the environment.

## Pathways for Transmission Between Animal and Human Resistomes

Several pathways allow exchanges between the animal and human microbiomes. Those most relevant for food-producing animals are directly from animal-to-man via the food chain (Aidara-Kane et al., 2013) or indirectly via the multiple intricate pathways of the environment [reviewed by (Ashbolt et al., 2013)].

**FIGURE 5:**
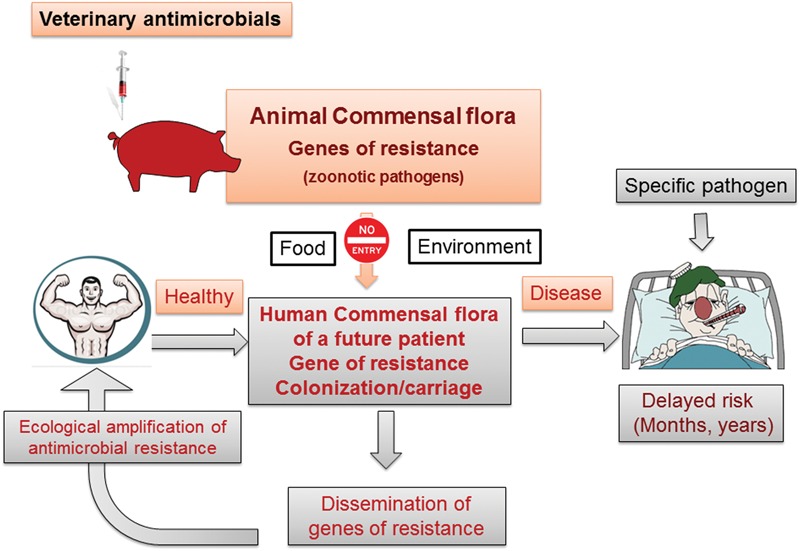
**Animal and human commensal microbiota illustrating the passage of resistance genes from animals to humans.** The acquisition in healthy subjects, either of resistance genes or resistant bacteria from food-producing animals, involves the pivotal role of the human commensal GIT microbiota. This can be regarded as a sieving filter, able first to retain and then amplify genes of resistance derived from the food chain or the environment. These persisting genes may subsequently be transmitted, possibly after a long delay, to specific human pathogens.

Scenarios for the transmission of veterinary AMR factors to humans are consistent with the hypothesis of a pivotal role of the human commensal microbiota in the natural history of human infections (van der Waaij and Nord, 2000; Andremont, 2003; Andremont et al., 2011; **Figure 5**). There are two major avenues for the emergence and spread of AMR in pathogenic bacteria in humans: the first is by direct selection of resistant mutants within the population of pathogenic organisms at the site of infection, followed by dissemination to a new patient by direct exposure, as occurs in confined hospital environments (epidemic pathway); the second is indirect, involving an initial selection of resistant bacteria in the commensal microbiota through horizontal transfer of resistance genes from non-pathogenic to pathogenic species and subsequent transfer of pathogenic bacteria, with a possible delayed effect on the host. Currently, the intestinal microbiota play a central role in the amplification, dissemination, and circulation of AMR between humans. This arises from diffusion into the community of enterobacteria producing extended spectrum β-lactamases (ESBL), predominantly of CTX-M type, produced by *E. coli*, which is ubiquitous in commensal microbiota. Thus, the human digestive tract can be viewed as an open door to AMR determinants from various external sources including food animals. Any subject (patient or not) is likely to be permanently exposed to resistance determinants (through the food chain, from the terrestrial and aquatic environments…) depending on the individual’s risk factors. These harmless bacteria and their resistance determinants are ingested and expose the GIT commensal microbiota, which can be viewed as a retention filter able to construct, for each individual, a personal pool of resistance genes, with the potential to subsequently undergo horizontal transfer to pathogens.

Transfer of resistance genes between *E. coli* residing in the human intestine was experimentally demonstrated in human volunteers (Trobos et al., 2009). Furthermore, the *in vivo* transfer of resistance genes from *E. faecalis* isolated from food of animal origin to a human isolate was demonstrated using a mouse model (Sparo et al., 2012). Finally, it was concluded from a mathematical model, evaluating factors affecting the prevalence of human commensal resistant bacteria causing opportunistic infections (e.g., enterococci), that the appearance of AMR bacteria in humans is hastened by the use of AMDs in agriculture (Smith et al., 2002).

## The Limitations of Pharmacodynamic Selectivity and the Requirement for Pharmacokinetic Selectivity

For AMDs, selectivity as generally understood, implies narrow spectrum of activity; for the prudent use of veterinary AMDs, narrow spectrum are preferred to broad spectrum agents (European Medicines Agency, 2015b). However, this recommendation relates to the target pathogen and pharmacodynamic selectivity alone cannot resolve public health considerations, because of the range of bacterial species, both Gram-positive and Gram-negative, in the commensal microbiota. For a sustainable veterinary AMD usage, the selectivity required is primarily pharmacokinetic and not pharmacodynamic. Thus, drug distribution in the body should ideally be restricted to the biophase (site of action) and, as a minimal requirement, excluded from the commensal GIT microbiota. The latter objective can be achieved either by selecting drugs not eliminated in the GIT or, if a drug is nevertheless present in active form in the distal GIT, by suppressing its activity through physical trapping or by favoring its local degradation into inactive compounds. An example is use of β-lactamases for β-lactam compounds [reviewed by (van der Waaij and Nord, 2000)].

Almost all currently used veterinary AMDs are eliminated into the GIT. For orally administered AMD, the principal factor is low bioavailability (see above) whilst for systemic administration the main factor is high biliary and/or intestinal clearance, both of which are associated with high drug lipophilicity. The physico-chemical properties of AMDs used in humans differ from those of other therapeutic classes. Human AMDs have a higher MW (371 ± 161 Da) and a lower lipophilicity (Calculated log octanol/water Partition coefficient, Clog P of -0.18 ± 1.88) than drugs of other classes. In addition, protein binding is relatively low for most AMDs and urinary excretion ratio relatively high (Sakaeda et al., 2001). In contrast, for AMDs used in food-producing animals, the position differs. For example, the 10 AMDs routinely used to treat bovine respiratory disease (O’Connor et al., 2013) have a higher MW (557 ± 220) and are more lipophilic (Clog P 1.8 ± 2.18) than those used in human medicine (**Table 1**).

**Table 1:** Relationship between lipophilicity and pharmacokinetic parameters for the 10 most used antimicrobial in cattle.

Antimicrobials	Classes	Molecular weight	XLogP3-AA	Vss (L/kg)	Clearance (mL/kg/min)	Half-life (h)
Ceftiofur	Cephalosporins	523.56	0.20	0.30	0.55	7.00
Danofloxacin	Fluoroquinolones	357.38	-0.30	2.48	8.30	4.01
Enrofloxacin	Fluoroquinolones	359.39	-0.20	1.80	3.20	6.60
Florfenicol	Amphenicols	358.21	0.80	0.77	3.75	2.65
Gamithromycin	Macrolides	777.03	4.90	24.90	11.86	44.90
Oxytetracycline	Tetracyclines	460.43	-1.60	0.79	1.88	5.66
Tildipirosin	Macrolides	734.01	4.30	21.80	2.40	238.00
Tilmicosin	Macrolides	869.13	3.60	28.20	11.40	28.00
Trimethoprim	Diaminopyrimidine	290.32	0.90	1.50	28.33	1.20
Tulathromycin	Macrolides	806.08	3.80	11.1	3.01	65
	Mean	556.89	1.80	10.37	8.24	40.30

*XlogP3 is the computed Octanol-Water Partition Coefficients (log P; Cheng et al., 2007) as given by PubMed ComPound; Vss: volume of distribution in steady-state condition. The 10 AMDs routinely used to treat bovine respiratory disease were listed by (O’Connor et al., 2013) they have a higher MW (557 ± 220) and are more lipophilic (log P = 1.8 ± 2.18) than those used in human medicine. The terminal half-life is positively correlated to the degree of lipophilicity with a coefficient of determination (R2) between lipophilicity and duration of half-of 0.37.*

**FIGURE 6:**
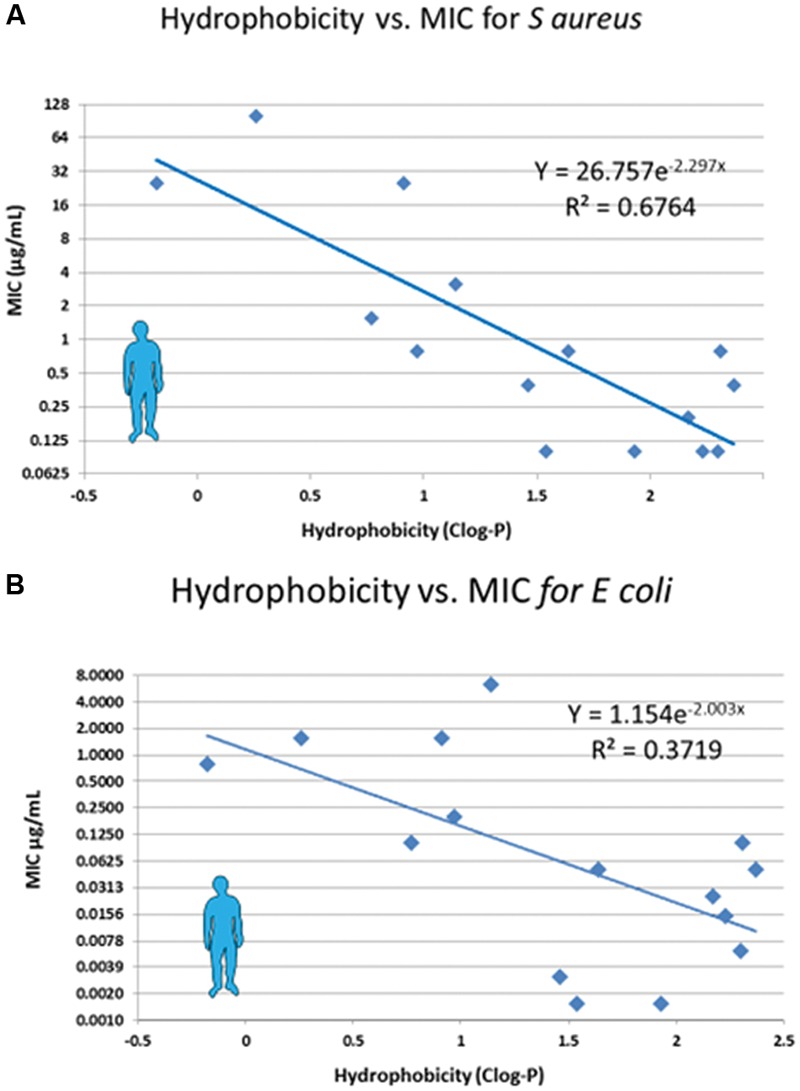
**Relationship between lipophilicity and potency of fluoroquinolones.** Potency of a series of fluoroquinolones was measured as the MIC against *Staphylococcus aureus* **(A)** and *E. coli* **(B)**; MIC data are from Takenouchi et al. (1996) and Clog P was obtained from PubChem Compound. As lipophilicity (and hydrophobicity) increase, so potency increases also.

Lipophilic drugs are selected for veterinary use for two reasons: first, the requirement for long duration of action, the terminal half-life being positively correlated to the degree of lipophilicity [the coefficient of determination (*R*^2^) for the 10 most used AMDs in cattle *R*^2^ = 0.37] (**Table 1**); and second, the need to develop more potent AMDs. The potency of quinolones in humans for *S. aureus* and *E. coli* correlates with lipophilicity (Clog P; **Figure 6**). Similarly, it would be valuable to assess the relationship between effectiveness of AMD (especially of quinolones and cephalosporins) to alter the GIT microbiota and their lipophilicity, because not all drugs of a given class are equivalent. The potential for developing more eco-friendly drugs recognizes that the most potent lipophilic AMDs are also likely the least selective in terms of tissue distribution. In fact, eco-friendly AMDs, such as ceftaroline (Panagiotidis et al., 2010) telavancin, dalbavancin (Nord and Edlund, 1990; van der Waaij and Nord, 2000; Edlung and Nord, 2003; Rashid et al., 2011, 2012) already exist in human medicine. This supports the feasibility of developing specific green AMDs in veterinary medicine. For this objective, it is necessary to recognize that a less potent drug is not synonymous with reduced clinical efficacy; it simply implies (other factors being equal in terms of pharmacological activity and clearance) a higher dosage regimen.

## Ideal Pharmacokinetic and Pharmacodynamic Profiles to Minimize Public Health Issues

**FIGURE 7:**
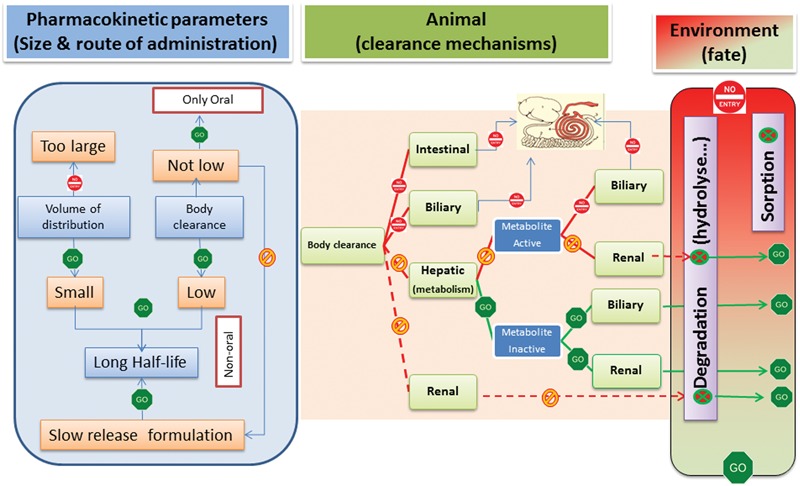
**Pharmacokinetic profile of the ideal veterinary green AMD.** The left panel indicates that the green AMD should not have a large volume of distribution. However, as volume of distribution is, with plasma clearance, one of two determinants of terminal half-life, an AMD with low volume of distribution will usually have too short a half-life. This is not a significant issue for the oral route, as an AMD with a short half-life can be administered virtually continually in feed or drinking water, in effect providing an oral infusion. In contrast, this is a major challenge for parenteral routes and development of slow release formulations (especially for AMDs exerting a time-dependent killing action) is necessary to ensure a long terminal half-life (flip-flop pharmacokinetics), thereby allowing both practicable and efficacious therapy with single dose administration. The middle panel indicates that a green AMD cannot have either intestinal or biliary clearance as a mechanism of elimination to ensure that there is no negative impact on the animal GIT microbiota. Clearance by hepatic metabolism is ideal, provided the metabolites are inactive. Alternatively, a high renal clearance is acceptable, provided the eliminated active drug is rapidly degraded in the environment or immobilized by physical sorption (right panel).

The ideal AMD for food-producing animals should possess several pharmacokinetic and pharmacodynamic characteristics to ensure practicability for use under field conditions, whilst having no detrimental impact on the GIT microbiota (**Figure 7**).

### High Oral Bioavailability

For oral dosing, a high bioavailability is required for two reasons: first, to minimize the unwanted impact on the GIT microbiota by the unabsorbed fraction; and secondly, because a high bioavailability is almost always associated with a lower between-animal variability (Toutain and Bousquet-Mélou, 2004a). Due to inter-animal variability, drug companies are constrained to selection of a high population dose to ensure efficacy for a high proportion of treated animals (usually the target attainment rate is 90%).

Development of AMDs with high oral bioavailability presents a significant challenge, as illustrated by the low oral bioavailability of currently used drugs in food producing animals. Studies comparing fasted and fed animals or before and after weaning demonstrated that feed (e.g., milk vs. hay) could have a large negative impact on oral bioavailability. This is likely attributable to the adsorption capacity of feed and especially of cellulose, a major constituent of animal feed (Ratz et al., 1995). Therefore, a green orally administered AMD should interact minimally with feed constituents.

For the unbound feed fraction, the ‘rule of five,’ also known as the Lipinski rule (Lipinski, 2000) provides guidance on factors controlling oral drug absorption; Lipinski’s ‘rule of five,’ is based on five descriptors of drug physico-chemical properties. The most important is lipophilicity, as quantified by the LogP (or Clog P). The degree of lipophilicity influences drug solubility and ability to permeate both intestinal epithelial cells and bacterial cell walls and membranes. Compounds that are too hydrophilic (having negative LogP) do not cross intestinal membranes and the ideal LogP is of the order of 2–3. Conversely, compounds with an *a priori* appropriate lipophilicity for oral absorption may be too lipophilic to penetrate bacteria. This is because drug penetration through the two bacterial membranes depends: first, on the balance between a hydrophilicity requirement (typically a negative LogP) to facilitate passage through water channels (aquaporins) tunneling outer bacterial membranes (especially in Gram-negative bacteria) and; second, a lipophilicity requirement to penetrate the inner lipid rich membrane, when the molecular target is intracellular, e.g., ribosomal bacterial enzymes (macrolides, tetracyclines…).

When the site of action is located between the two membranes, as for β-lactams or colistin-like drugs, the requirement is for more hydrophilic compounds. There is a trade-off between lipophilicity for optimal oral bioavailability and hydrophilicity for optimal bacterial penetration; most AMDs in veterinary use do not conform to the stipulations of the rule of five (Lipinski et al., 2001). The development of green AMDs should take into account that less lipophilic (more hydrophilic) compounds are required than other drug classes. To resolve this dual requirement, a pro-drug approach is an attractive option; a relatively lipophilic and well absorbed pro-drug, followed by first-pass hepatic metabolism to generate an active, more hydrophilic, metabolite. This strategy was deployed effectively for pro-drugs of ampicillin (LogP = 0.57) such as bacampicillin (LogP = 2.04) and pivampicillin (LogP = 2.4).

### Development of Products (Substances or Formulations) Ensuring Persistency of Effective Plasma Concentrations

In ruminating cattle and some swine production systems, intramuscular, and subcutaneous administration routes are extensively used. Formulations with high systemic bioavailability are readily achieved and there is no direct exposure of the GIT microbiota, as with the oral route. However, parenteral administration must allow for an appropriate duration of activity. In contrast to oral dosing, the requirement is for long-acting products to provide bacteriological cure with single dose administration. Such products avoid fluctuating plasma and biophase drug concentrations and are desirable also from economic and animal welfare perspectives.

For cattle bronchopneumonia therapy, the ideal activity duration is typically at least 5 days. Persistence of action can be achieved with several strategies, which have differing consequences in terms of bacteriological selectivity. One option is to develop a drug substance with a long intrinsic elimination half-life, as for most macrolides used in food-producing animals. Half-life is a hybrid parameter controlled by plasma clearance and drug distribution in peripheral compartments. Therefore, a long half-life can be obtained either by selecting compounds with a low clearance or, alternatively, by selecting those with a large volume of distribution. These strategies are not equivalent in terms of pharmacokinetic selectivity. Thus, AMDs with a large volume of distribution cannot be selective, because they are extensively distributed outside the extracellular water space, while most pathogens of veterinary interest are located in this space. Moreover, a large distribution volume is generally associated with high lipophilicity (Yusof and Segall, 2013) and such drugs are excreted in the GIT, with possible unwanted effects on the GIT microbiota. Alternatively, a long half-life can be obtained if the drug has a low plasma clearance. It is plasma clearance, not volume of distribution, which controls steady-state plasma drug concentrations, and hence concentration in the biophase (Toutain and Bousquet-Mélou, 2004b). In summary, the optimal strategy in developing a long-acting substance with the required pharmacokinetic selectivity (located primarily in the extracellular space) is to select candidates with low clearance rather than a large volume of distribution. The likelihood of achieving a low distribution volume at steady state (Vss) decreases below average if the logP is greater than approximately one (Yusof and Segall, 2013). Of course, this strategy will not be suitable for intracellular infections (e.g., caused by mycoplasmas); these require some lipophilicity to penetrate intracellularly.

An alternative approach to minimize impact on GIT microbiota is to develop long-acting formulations, rather than long-acting compounds, with eco-friendly properties. The selected compound would have a limited distribution volume, appropriate clearance and short elimination half-life, but effective concentrations are ensured through slow but maintained absorption from the injection site, giving rise to flip-flop pharmacokinetics (Toutain and Bousquet-Melou, 2004). These new formulations for green AMDs must be locally well tolerated (non-irritant) and administered as a single dose.

### Preferential Renal Clearance to Avoid GIT Microbiota Exposure

The principal routes of AMD elimination are hepatic and renal; hepatic clearance can comprise both metabolic inactivation and biliary secretion. Drugs may also be excreted directly into the GIT lumen by intestinal clearance.

According to (Kusama et al., 2010) the major pathways of drug clearance can be predicted *in silico* from knowledge of four physico-chemical parameters (charge, MW), lipophilicity and unbound protein fraction in plasma). Each is defined by and can be deduced from the drug’s molecular structure.

Preferential renal elimination is highly desirable, because this route is not associated with direct exposure of the GIT commensal microbiota. Also ideal would be renal elimination as inactive metabolites, preceded by phase I hepatic metabolism, to avoid any effect on the environmental microbiome. The relationship between physico-chemical properties of drugs and urinary excretion has been documented (Ito et al., 2013). Drugs that are eliminated unchanged by the kidneys have a small MW, large unbound fraction and are hydrophilic.

### Avoiding Elimination by Biliary or Intestinal Clearance to Protect the GIT Microbiome

On the Biopharmaceutics Classification System (Amidon et al., 1995) drugs are classified as highly or poorly permeable, according to their rate of transit across intestinal cells. Poorly permeable drugs generally require an active transport process, involving eﬄux transporters, to cross cell membranes efficiently and many veterinary AMDs and/or their active metabolite(s) are eliminated into the GIT either by active biliary secretion or by intestinal clearance. Therefore, potential new drugs should be screened for a lack of affinity for the biliary and GIT eﬄux pumps to ensure no exposure of commensal GIT microbiota. Lipophilicity and MW are the two most important determinants for biliary excretion and reliable screening procedures exist for predicting drug elimination in bile (Rioux et al., 2013). For intestinal eﬄux, *P*-glycoprotein is a well characterized ABC transporter. It limits the oral bioavailability of poorly soluble drugs but also extrudes some substances by intestinal clearance. Fluoroquinolones are substrates for multiple human ABC transporters (Alvarez et al., 2008) as demonstrated for enrofloxacin, ciprofloxacin and danofloxacin. *P*-glycoprotein substrates tend to have higher lipophilicity and/or larger MW than non-substrates. *In silico* models for predicting the probability that a compound will interact with *P*-glycoprotein or analogous transporters are available and could be used to select candidates for green AMDs (Chen et al., 2012; Montanari and Ecker, 2015).

## Disposition Properties to Minimize Environmental Hazard

A green antibiotic should not be eliminated into the environment as an active substance. If, nevertheless, a potential candidate is excreted in urine as parent drug or active metabolites, their fate, and any ongoing activity in the various environmental matrices, should be determined. The possibility of optimizing degradability in waste must be considered. Because AMDs generally reach the environment via water eﬄuent from livestock operations, hydrolysis is a potentially important degradation pathway. β-lactams, macrolides, and sulfonamides are three classes susceptible to hydrolysis (Adams et al., 2013), whereas little or no degradation was reported for oxytetracycline (Jacobsen and Berglind, 1988).

Photolysis is also an abiotic transformation process; in addition, photodegradation can occur at the soil-atmosphere interface and on the surface of liquid manure. Quinolones and tetracyclines are susceptible to photodegradation (Huang et al., 2011) and photodecomposition of AMDs under field conditions is a desirable property.

Possible interactions of non-degraded AMD fractions with soil dwelling bacteria should be explored (Subbiah et al., 2011). This can be accomplished by mixing test antibiotics or their active metabolites with soil slurries differing in clay content, pH, and other properties. After mixing, the supernatant can be assayed with a sensitive *E. coli* strain that is only inhibited by the freely available AMD fraction, i.e., the mobile fraction not adsorbed by soil materials (Subbiah et al., 2011). This type of assay showed that tetracyclines, neomycin, and ciprofloxacin were not mobile whereas florfenicol and β-lactams were mostly available in the liquid phase and therefore able to affect the test bacteria. This can be also predicted from the organic normalized dissociation constants (Koc) of candidate compounds. The Koc provides, although imperfectly, a prediction of how firmly AMDs adsorb to soil (Tolls, 2001). In addition, sorption coefficients have been proposed to predict AMD mobility in the environment (Call et al., 2013).

## Regulatory Considerations

The provision of incentives to develop innovative AMDs requires a favorable regulatory climate. All regulatory authorities (EMA, FDA/EPA…) must consider the case of green antimicrobials as alternatives to currently available drugs, as this is essential to fulfill the main requirement of the One health policy, namely to be lacking in side-effects for human health and environment. To promote green AMDs, and to give them a competitive advantage over conventional AMDs, regulatory authorities will need to consider the impact of all AMDs (new or old) on the environmental resistome, before granting a marketing authorization. Currently, the issue of AMR transmitted by environmental pathways is not considered in guidelines either for generic or pioneer products.

A similar situation exists in the USA and it was recently suggested that EPA “*should explore requiring FIFRA (Federal Insecticide, Fungicide, and Rodenticide Act) registration of antibiotics used in food animal production. Data requirements for registration will help focus and support the restricted use that is important for deterring selection of antibiotic resistance in the food supply, and in the environment due to farm antibiotic eﬄuents*” (Metz and Shlaes, 2014). Recently, in the EU, CVMP explicitly acknowledged “*the importance of the environment as a reservoir for AMR genes*” (European Medicines Agency, 2015a) and “*the CVMP will develop a reflection paper to consider the role of AMR in the environment and the feasibility of addressing this in the environmental risk assessment for veterinary medicinal products.*”

Regulatory authorities will need to obtain and assess data to facilitate the development of green antibiotics. Examples are: the provision of robust PK/PD data; confirmation of efficacy in non-inferiority clinical trials and the submission of data which clearly demonstrates ecological advantages over conventional comparators.

According to some economic models, AMR can be reduced by extending the duration of the patent on pioneer AMDs, because patents give the owners incentives to protect the value of their drugs by limiting usage (Horowitz and Moehring, 2004). The pricing of green antibiotics should reward industry for costly R&D efforts but this will be difficult to achieve if the market is dominated by inexpensive older products (both generic and pioneer products). Prescribing more expensive green antibiotics, whose advantage is not to enhance therapeutic outcome but rather to address public health issues, would be less likely under conditions of farm animal practice.

An important consideration is the expression of AMD consumption in terms of mass units. Green AMDs would likely be less potent than their available counterparts and therefore would require a higher dosage. Consideration should therefore be given to adoption of alternative units of measurement, when reporting antimicrobial consumption data in food-producing animals. This may be necessary to avoid penalizing green antibiotics and to ensure a less biased expression system of AMD consumption (Dewulf et al., 2013). Alternatives have been adopted in the Netherlands (Taverne et al., 2015) and Denmark and also in human medicine, where the WHO system of harmonized Defined Daily Dosages is used.

## Conclusion

Veterinary AMDs, in the longer term, should be innovative and expensive, and their marketing strictly regulated, to ensure minimal public health impact.

The main thesis proposed in this article is that we urgently need new AMDs in veterinary medicine, because most of the drugs currently used ineluctably expose the animal GIT microbiome, through their lack of pharmacokinetic selectivity. Thereby, they potentially help to enrich the human resistome, i.e., increase accumulation of AMD resistance genes harbored by both pathogenic and non-pathogenic human bacteria. The development of green AMDs is based on the consideration that AMR of veterinary origin should be viewed as a global ecological challenge and not as a medical issue and that solutions need to consider the eco-evo rationale as outlined by (Baquero et al., 2011). For new and innovative green antibiotics the pivotal characteristic will be minimal ecological impact. They will possess appropriate PK/PD selectivity, in that they will be distributed only or primarily in the biophase where the targeted pathogen is located. Consequently, they will have no negative impact either on the commensal GIT microbiota of the treated animal or on the various matrix/ecosystems of the environment. It should be possible to achieve these aims by re-evaluating the current classes of veterinary AMDs and applying the wealth of existing knowledge of medicinal chemistry to develop more hydrophilic analogs of these drug. Hence, there should be no requirement to discover and develop wholly new drug classes, with novel mechanisms of action that indeed might be challenged on the ground of new additional AMR risks for human medicine.

## Author Contributions

All authors listed, have made substantial, direct and intellectual contribution to the work, and approved it for publication.

## Conflict of Interest Statement

The authors declare that the research was conducted in the absence of any commercial or financial relationships that could be construed as a potential conflict of interest.

## Abbreviations

AMD: antimicrobial drugs
AMR: antimicrobial resistance
AUC: area under the curve
CVMP: Committee for Medicinal Products for Veterinary Use
EMA: European Medicine Agency
GIT: gastro intestinal tract
MIC: minimum inhibitory concentration
MW: molecular weight
PK/PD: pharmacokinetic/pharmacodynamic
